# Fossil lemurs from Egypt and Kenya suggest an African origin for Madagascar’s aye-aye

**DOI:** 10.1038/s41467-018-05648-w

**Published:** 2018-08-21

**Authors:** Gregg F. Gunnell, Doug M. Boyer, Anthony R. Friscia, Steven Heritage, Fredrick Kyalo Manthi, Ellen R. Miller, Hesham M. Sallam, Nancy B. Simmons, Nancy J. Stevens, Erik R. Seiffert

**Affiliations:** 1 Division of Fossil Primates, Duke Lemur Center, Durham, NC 27705 USA; 2 Department of Evolutionary Anthropology, Duke University, Durham, NC 27705 USA; 3 Department of Integrative Biology & Physiology, University of California – Los Angeles, Los Angeles, CA 90095 USA; 4 Interdepartmental Doctoral Program in Anthropological Sciences, Stony Brook University, Stony Brook, NY 11794 USA; 5 Department of Earth Sciences, National Museums of Kenya, Museum Hill, P.O. Box 40658-00100, Nairobi, 00100 Kenya; 6 Department of Anthropology, Wake Forest University, Winston-Salem, NC 27106 USA; 7 Mansoura University Vertebrate Paleontology Center (MUVP), Department of Geology, Faculty of Science, Mansoura University, Mansoura, 35516 Egypt; 8 Division of Vertebrate Zoology, Department of Mammalogy, American Museum of Natural History, New York, NY 10024 USA; 9 Department of Biomedical Sciences, Heritage College of Osteopathic Medicine, Ohio University, Athens, OH 45701 USA; 10 Center for Ecology and Evolutionary Studies, Ohio University, Athens, OH 45701 USA; 11 Department of Integrative Anatomical Sciences, Keck School of Medicine of USC, University of Southern California, 1333 San Pablo Street, BMT 406, Los Angeles, CA 90033 USA; 12 Department of Mammalogy, Natural History Museum of Los Angeles County, Los Angeles, CA 90007 USA

## Abstract

In 1967 G.G. Simpson described three partial mandibles from early Miocene deposits in Kenya that he interpreted as belonging to a new strepsirrhine primate, *Propotto*. This interpretation was quickly challenged, with the assertion that *Propotto* was not a primate, but rather a pteropodid fruit bat. The latter interpretation has not been questioned for almost half a century. Here we re-evaluate the affinities of *Propotto*, drawing upon diverse lines of evidence to establish that this strange mammal is a strepsirrhine primate as originally suggested by Simpson. Moreover, our phylogenetic analyses support the recognition of *Propotto*, together with late Eocene *Plesiopithecus* from Egypt, as African stem chiromyiform lemurs that are exclusively related to the extant aye-aye (*Daubentonia*) from Madagascar. Our results challenge the long-held view that all lemurs are descended from a single ancient colonization of Madagascar, and present an intriguing alternative scenario in which two lemur lineages dispersed from Africa to Madagascar independently, possibly during the later Cenozoic.

## Abstract (web summary)

The fossil taxon *Propotto* was originally identified as a primate, but is currently widely interpreted as a bat. Here, the authors identify *Propotto* as a stem chiromyiform lemur and, based on phylogenetic analysis, suggest two independent lemur colonizations of Madagascar.

## Introduction

Strepsirrhine or “toothcombed” primates include three ancient clades that diverged early in the Paleogene—Chiromyiformes (represented by one living and one subfossil species of aye-aye, both placed in the genus *Daubentonia*), Lemuriformes (containing extant lemurids, indriids, cheirogaleids, and lepilemurids, as well as the recently extinct archaeolemurids, palaeopropithecids, and megaladapids^1^), and Lorisiformes (lorisids and galagids). Recent molecular divergence estimates suggest that Madagascar’s lemurs (the clade containing both chiromyiforms and lemuriforms) split from lorisiforms in the Paleocene or early Eocene^1–5^, with lemurs colonizing Madagascar and then rapidly splitting into chiromyiform and lemuriform lineages^1–3^. The absence of a terrestrial Paleogene or Neogene fossil record on Madagascar^6^ has prevented paleontologists from testing this hypothesis. Given its geographic proximity to Madagascar, the adjacent African landmass is currently viewed as the most likely source for ancestral lemurs (and other endemic terrestrial mammals of Madagascar)^6–8^. Indeed, Paleogene ocean currents have been reconstructed as favoring west-to-east dispersal across the Mozambique Channel^9^, and together with the presence of both stem strepsirrhines and lorisiforms in Africa’s Eocene fossil record^10–12^ support this interpretation.

The primary challenge to the hypothesis of a single colonization of Madagascar by lemurs is the morphological evidence provided by the fossil primate *Plesiopithecus teras*, represented by a partial cranium and multiple mandibles from a single ~34 Ma (terminal Eocene) site in Egypt^13,14^. Godinot^15^ suggested a relationship between *Plesiopithecus* and extant *Daubentonia*, noting that “[*Plesiopithecus*’] lower jaw has exactly the morphology that would be predicted for a daubentoniid ancestor, having already markedly enlarged its anterior tooth and reduced the teeth posterior to it” (p. 457). Although marked enlargement of a single highly procumbent anterior lower tooth occurred more than once in primate evolution (e.g., in Eocene omomyiform haplorhines^16^), it is nevertheless an exceptionally rare pattern, and among extant primates is now seen only in *Daubentonia*, a taxon with highly specialized rodent-like anterior teeth. Godinot’s phylogenetic hypothesis linking *Daubentonia* and *Plesiopithecus* depends heavily upon their enlarged anterior lower teeth being homologous, as the cheek teeth of *Daubentonia* are highly modified and bear little resemblance to those of any living or extinct primate; the anterior teeth of *Daubentonia* are likely incisors^17^, whereas those of *Plesiopithecus* could be either canines or incisors^18^. Although Godinot did not test his hypothesis with an algorithm-driven phylogenetic analysis, it recently gained some support from a Bayesian tip-dating analysis of morphological and molecular data^19^ that, for some treatments of the morphological data, recovered evidence for a *Daubentonia-Plesiopithecus* clade within lemurs, despite the fact that, following previous interpretations^13,20^, the enlarged anterior lower tooth of *Plesiopithecus* was scored as a canine rather than as an incisor.

Here we present several new lines of evidence suggesting that the purported pteropodid fruit bat *Propotto* from the early Miocene of western Kenya is not only a strepsirrhine primate as was originally suggested by Simpson^21^, but represents a close relative of both *Plesiopithecus* and *Daubentonia*. *Plesiopithecus* was first described in 1992^14^, a quarter century after the debate surrounding *Propotto*’s affinities appeared to have been resolved, hence its significance for interpreting *Propotto* was not appreciated. Our comparisons indicate that *Propotto* shares a number of specialized morphological features with *Plesiopithecus*, including all of the features that originally led Walker^22^ to doubt Simpson’s proposed lorisid affinities for *Propotto*. Furthermore, digital reconstruction of the badly damaged upper molars of *Plesiopithecus* and comparisons with other specimens led us to the identification of two upper molars of *Propotto*, previously identified as “Lorisidae indet.”^23^, from the early Miocene site of Chamtwara, Kenya. These specimens offer the first glimpse of *Propotto*’s upper dentition and further buttress the case for its strepsirrhine primate affinities. Finally, a mandible of *Plesiopithecus* is here interpreted as retaining a small toothcomb-like canine distal to its enlarged anterior tooth, establishing that the enlarged anterior tooth is probably an incisor, thereby increasing the likelihood that the procumbent anterior lower teeth of *Daubentonia*, *Propotto*, and *Plesiopithecus* are homologous incisors.

## Results

### Comparative dental morphology of *Propotto* and *Plesiopithecus*

**Fig. 1:**
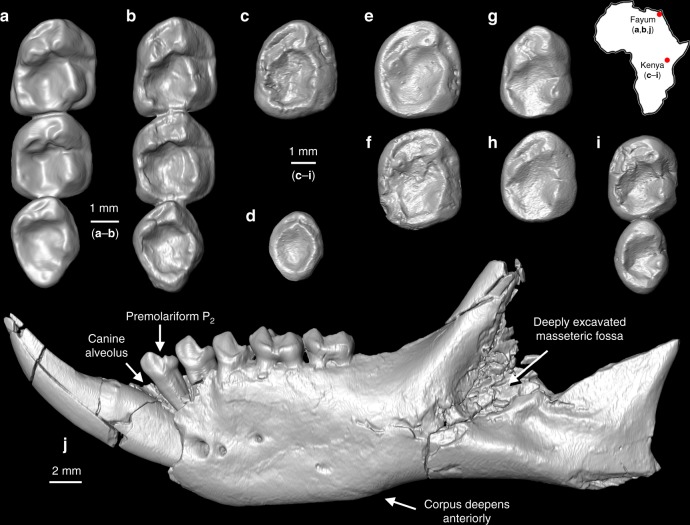
Comparison of lower molar morphology of latest Eocene *Plesiopithecus teras* and early Miocene *Propotto leakeyi* and mandibular morphology and lower dentition of *Plesiopithecus teras*. **a** M_1–3_ of DPC 11636, left mandible of *Plesiopithecus teras* (reversed for comparison, latest Eocene, Quarry L-41, Fayum Depression, Egypt); **b** M_1–3_ of CGM 42291, holotype right mandible of *Plesiopithecus teras*; **c** Left M_1_ of KNM-RU 1879, mandible of *Propotto leakeyi* (reversed for comparison; Simpson’s specimen “S”; note that this specimen is probably from Songhor despite the Rusinga accession number); **d** Left M_3_ of KNM-RU 1879, mandible of *Propotto leakeyi*, reversed for comparison; **e** KNM-CA 1832, isolated right M_1_ of *Propotto leakeyi* (early Miocene, Chamtwara, Kenya); **f** KNM-CA 2195, isolated right M_2_ of *Propotto leakeyi* (early Miocene, Chamtwara, Kenya); **g** M_1_ of KNM-SO 508, holotype right mandible of *Propotto leakeyi* (early Miocene, Songhor, Kenya; Simpson’s specimen “R”); **h** M_2_ of KNM-SO 508, holotype right mandible of *Propotto leakeyi*; **i** M_2–3_ of KNM-RU 2084, right mandible of *Propotto leakeyi* (possibly from Songhor despite the Rusinga accession number; Simpson’s specimen “T”); **j** DPC 13607, left mandible of *Plesiopithecus teras*, with an alveolus that we interpret as being for a small canine, and tooth crowns that we interpret as I_1_ or I_2_ and P_2_-M_2_. Digital models were created using CT scans made available by the Duke Lemur Center Division of Fossil Primates and the National Museums of Kenya, which were downloaded from www.morphosource.org and made available for reuse under a CC BY-NC license. Map of Africa is adapted from Google Earth

**Fig. 2:**
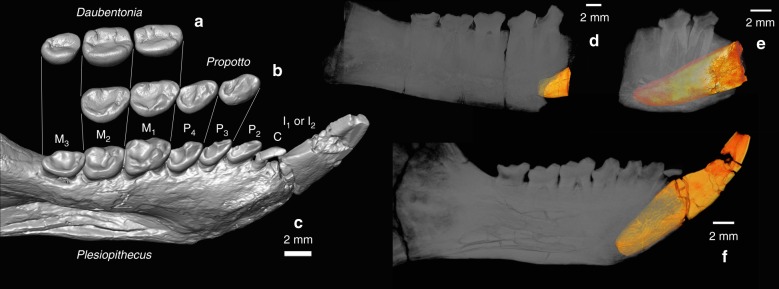
Comparison of lower molar morphology of *Daubentonia*, *Plesiopithecus*, and *Propotto*, and volume rendering of the enlarged anterior teeth of *Plesiopithecus* and *Propotto*. **a** Left M_1–3_ of AMNH M-41334, extant *Daubentonia madagascariensis*, with individual surfaces reoriented slightly to facilitate comparison (teeth are from right side in AMNH M-41334 but are reversed for comparison); **b** left P_3_-M_2_ of *Propotto leakeyi* (holotype mandible KNM-SO 508, teeth are from right side but are reversed and reoriented slightly to facilitate comparison); **c** left mandible with I_1_ or I_2_ and canine-M_3_ of *Plesiopithecus* (DPC 11636). Scale in left panel is for **a**–**c** (2 mm). **d**–**f** Volume renderings of the enlarged anterior tooth (probable I_1_ or I_2_, rendered orange-yellow) in **d** KNM-KO 101, left mandible with partial root of I_1_ or I_2_ and crowns of P_3_-M_2_, *Propotto leakeyi* (early Miocene, Koru, Kenya); **e** KNM-RU 3690, right mandibular fragment with root and partial crown of I_1_ or I_2_ and crowns of P_3–4_, cf. *Propotto leakeyi* (early Miocene, Rusinga Island, Kenya); note that in this specimen the root of I_1_ or I_2_ extends under the roots of M_1_; **f** DPC 11636, left mandibular corpus with complete crowns of I_1_ or I_2_ and canine-M_3_, *Plesiopithecus teras*. Digital models were created using CT scans made available by the Duke Lemur Center Division of Fossil Primates and the National Museums of Kenya, which were downloaded from www.morphosource.org and made available for reuse under a CC BY-NC license

*Propotto leakeyi* was originally described by Simpson as a lorisiform strepsirrhine that might be related to the extant lorisid *Perodicticus* (commonly known as the potto)^21^. The hypodigm available to Simpson included the holotype (KNM-SO 508, his specimen “R”; KNM = National Museums of Kenya), a right mandible with P_3_-M_2_ and alveoli for P_2_ and M_3_ as well as a small portion of the root of an enlarged anterior tooth (Figs. 1g and 2b); KNM-RU 1879 (specimen “S”), a left mandible with a very shallow P_2_ alveolus, an erupting P_3_, fully erupted dP_4_ and M_1_, alveoli for M_2_ and an erupting M_3_ (see M_1_ and M_3_ in Fig. 1c, d, respectively; this specimen also exhibits a laterally compressed and forward-facing alveolus for an anterior tooth); and KNM-RU 2084 (specimen “T”), a right mandible with M_2–3_ that is most likely from Songhor but labeled as being from Rusinga (Fig. 1i). KNM-RU 1879 has “Songhor” written on the specimen despite the fact that the label suggests it might be from Rusinga; we consider it probable that the specimen is, in fact, from Songhor. If all of these specimens are indeed from Songhor, they would originate in the Chamtwara and the “Kapurtay Conglomerates” of Butler^24^, which Pickford^25^ put in his “Set I” fauna, and dated at 18.5–20 Ma. This estimate is mainly based on K-Ar dates of Bishop et al.^26^ published in 1969, so additional work is needed to provide more precise age constraints for these localities using contemporary methodologies. Digital models of all the fossil specimens figured here are available on MorphoSource (Table 1).

**Table 1:** DOI addresses for digital surface models of figured fossil specimens

Taxon	Specimen	DOI	Element scanned
*Karanisia clarki*	DPC 21636E	doi.org/10.17602/M2/M39083	Right M^2^
*Karanisia clarki*	DPC 21639C	doi.org/10.17602/M2/M39082	Right M^1^
*Plesiopithecus teras*	CGM 42291	doi.org/10.17602/M2/M39088	Right mandible (cast)
*Plesiopithecus teras*	DPC 11636	doi.org/10.17602/M2/M31629	Left mandible
*Plesiopithecus teras*	DPC 12393	doi.org/10.17602/M2/M38310	Cranium
*Plesiopithecus teras*	DPC 13607	doi.org/10.17602/M2/M38308	Left mandible
*Propotto leakeyi*	KNM-CA 1796	doi.org/10.17602/M2/M39085	Right M^2^ (cast)
*Propotto leakeyi*	KNM-CA 1797	doi.org/10.17602/M2/M39084	Right M^1^ (cast)
*Propotto leakeyi*	KNM-CA 1832	doi.org/10.17602/M2/M39092	Right M_1_ (cast)
*Propotto leakeyi*	KNM-CA 2195	doi.org/10.17602/M2/M39095	Right M_2_ (cast)
*Propotto leakeyi*	KNM-KO 101	doi.org/10.17602/M2/M26191	Left mandible
*Propotto leakeyi*	KNM-RU 1879	doi.org/10.17602/M2/M26200	Left mandible
*Propotto leakeyi*	KNM-RU 2084	doi.org/10.17602/M2/M33617	Right mandible (cast)
*Propotto leakeyi*	KNM-RU 3690	doi.org/10.17602/M2/M26203	Right mandible
*Propotto leakeyi*	KNM-SO 508	doi.org/10.17602/M2/M33601	Right mandible (cast)

Simpson^21^ noted the “highly peculiar cheek teeth” (p. 51) of *Propotto* and the fact that its mandible deepened anteriorly, but nevertheless considered this taxon to be similar enough to the extant lorises *Perodicticus* and *Nycticebus* to recognize *Propotto* as an aberrant lorisid. Walker^22^ re-examined the hypodigm of *Propotto* and pointed out that the single-rooted P_2_ was probably small (though no crown is preserved), and not enlarged and caniniform as in lorisiforms. Further, he interpreted the alveolus of *Propotto*’s enlarged anterior lower tooth as being for a caniniform canine, and contrasted that with the canine morphology that would be expected in lorisiforms, which incorporate the canine into a toothcomb. Finally, he noted that the mandibular corpus was also unlike those of lorisiforms in deepening anteriorly and having a deep masseteric fossa. Walker concluded that *Propotto* was a pteropodid fruit bat and not a primate, a conclusion that was accepted by Simpson in correspondence exchanged before the 1969 publication of Walker’s work (Supplementary Fig. 1).

In 1984, Butler^24^ described a few additional *Propotto* specimens from the early Miocene sites of Koru, Chamtwara, and Rusinga Island in western Kenya^25^. The specimens from Rusinga localities located in the Hiwegi Formation would be considerably younger, dated to ~17.9 Ma^27^. Butler discussed the resemblance of *Propotto*’s cheek teeth to those of primates such as *Cheirogaleus*, *Perodicticus*, and *Pithecia*, and also with the Neotropical phyllostomid bat *Artibeus*. He further noted that the enlarged anterior lower tooth of *Propotto* (which he also interpreted as a canine) is relatively larger than the lower canines of extant pteropodids, having a root that extends posteriorly to at least P_3_. Despite these observations, Butler ultimately supported the idea that *Propotto* represented a side-branch of the chiropteran family Pteropodidae, erecting a new subfamily, Propottininae, for the genus.

For the last half-century, discussion of *Propotto*’s significance as a possible primate has been deterred by the authoritative consensus reached by Simpson, Walker, and Butler that *Propotto* is a bat. However, it is now clear that the features that Walker cited in his criticism of Simpson’s identification of *Propotto* as a lorisid are all characteristic of the undoubted strepsirrhine primate *Plesiopithecus* and so do not necessarily exclude *Propotto* from Strepsirrhini (Fig. 1j). The laterally compressed and presumably highly procumbent lower anterior tooth of *Propotto* (Fig. 2d, e; unknown to both Simpson and Walker because this feature is only preserved in specimens described by Butler in 1984) does not occur in fruit bats, or for that matter any known living or extinct chiropteran. This feature is, however, present in *Plesiopithecus* (Figs. 1j and 2d–f) and *Daubentonia*.

**Fig. 3:**
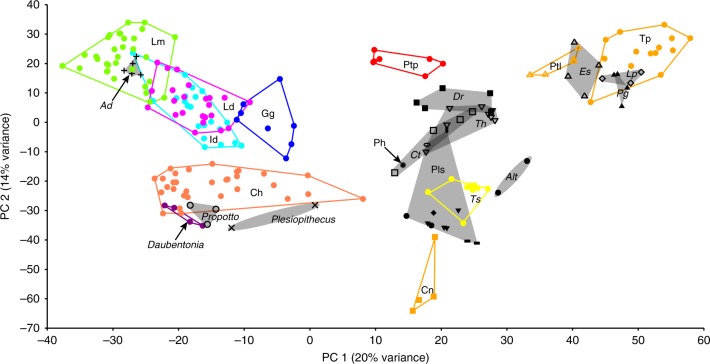
First two principal components (PC) resulting from principal component analysis of 1100 pseudolandmarks on a broad taxonomic sample of second lower molar teeth. Each point represents the tooth of one individual. Convex hulls and different colors indicate distinct extant taxonomic groups. Gray hulls with different marker symbols represent different fossil taxa. This sample includes teeth of extant non-primate treeshrews (Ptl: *Ptilocercus*, Tp: *Tupaia* sp.), cynocephalid dermopterans (Cn), and pteropodid fruit bats (Ptp). It also includes fossil non-primates and possible stem primates including *Leptacodon* sp. (*Lp*), various plesiadapiforms (Pls), and *Altanius* (*Alt*). It includes a number of extant primates including strepsirrhine lemurids (Lm), indriids (Id), cheirogaleids (ch), lorisids (Ld), and galagids (Gg), as well as *Daubentonia madagascariensis*. The extant haplorhine *Tarsius* is also included (Ts). Fossil haplorhines include *Eosimias* (*Es*) and *Phenacopithecus* (*Ph*). Early fossil prosimians include *Donrussellia* sp. (*Dr*), *Cantius torresi* (*Ct*), and *Teilhardina* sp. (*Th*). See Supplementary Data 1 for a list of all specimens included. See Supplementary Data 2 and 3 for principal component scores of additional components (e.g., 3–4), which also support the groupings of PC1–2

Despite being very low-crowned, the lower molars of *Propotto* are fundamentally strepsirrhine in structure, and are very similar to those of *Plesiopithecus* (Fig. 1a–i). Differences from *Plesiopithecus* include extension of the oblique cristids to meet the protoconid apices, reduction or elimination of hypoflexids, reduction of metaconids, and presence of a cingulid around the lingual margin of the metaconids. The P_3–4_ of *Propotto* and *Plesiopithecus* are very similar in having mesially shifted protoconids from which two dominant crests run distobuccally and distolingually to enclose well-developed talonids (Fig. 2b, c). An automated geometric morphometric analysis of lower molar morphology in strepsirrhines, pteropodids, *Propotto*, and various living and extinct euarchontans demonstrates that the shape of *Propotto*’s M_2_ is most similar to that of strepsirrhines (and particularly those of cheirogaleids, *Daubentonia* and *Plesiopithecus*; Fig. 3). The molar morphology of this set of taxa occupies a morphospace that is distinct from sampled pteropodids. Strepsirrhine lower molars also have low principal component (PC) 1 values that separate them from all non-euarchontans, non-primates, tarsiers, and almost all sampled Paleogene primates. The only fossils that group with modern strepsirrhines on PC1 are *Adapis*, *Propotto*, and *Plesiopithecus*. On PC2, strepsirrhines are divided into a cluster of lemurids, indriids, lorisiforms, and *Adapis* with high values, and another including cheirogaleids (*Microcebus, Cheirogaleus, Mirza, Phaner*), *Daubentonia*, *Plesiopithecus*, and *Propotto* with low values. The pteropodid fruit bats *Pteropus* and *Rousettus* are well-separated from *Propotto* in having much higher PC1-2 scores, although they do overlap with the second group of strepsirrhines. Although we only plot PC1 (20% of variance) and PC2 (14% of variance) in Fig. 3, the most important clustering patterns are maintained on PC3 and PC4, as well (Supplementary Data 1–3).

**Fig. 4:**
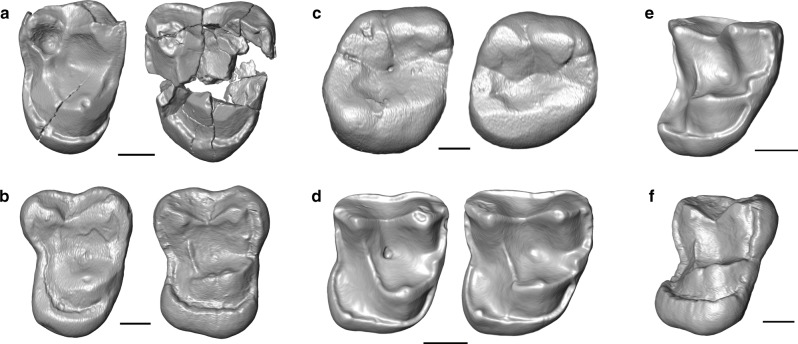
Upper molars of early African strepsirrhines and extant *Daubentonia* from Madagascar. **a** Left M^1^ (on right) and M^2^ (on left) of DPC 12393, partial cranium of *Plesiopithecus teras* (latest Eocene, Quarry L-41, Fayum Depression, Egypt); reversed for comparison, the M^1^ is badly damaged and has been digitally reconstructed by segmenting out multiple fragments and repositioning them, while the M^2^ is lacking much of the buccal margin; **b** isolated right M^1^ [KNM-CA 1796, on right] and M^2^ [KNM-CA 1797, on left] of *Propotto leakeyi* (early Miocene, Chamtwara, western Kenya); **c** right M^1^ (on right) and M^2^ (on left) of AMNH M-41334, adult *Daubentonia madagascariensis* individual from Madagascar, locality unknown; **d** right M^1^ [on right, DPC 21639C] and M^2^ [on left, DPC 21636E] of *Karanisia clarki* (earliest late Eocene, Quarry BQ-2, Fayum Depression, Egypt); **e** oblique mesial view of DPC 21639C, right M^1^ of earliest late Eocene *Karanisia clarki*, showing the tall primary cusps, low parastyle, low lingual cingulum, and paraconule typical of early strepsirrhines; **f** oblique mesial view of KNM-CA 1796, right M^1^ of *Propotto leakeyi*, showing the low primary cusps, relatively tall parastyle, tall lingual cingulum, and absence of paraconule that is characteristic of this species. Scale is equal to 1 mm. Digital models were created using CT scans made available by the Duke Lemur Center Division of Fossil Primates and the National Museums of Kenya, which were downloaded from www.morphosource.org and made available for reuse under a CC BY-NC license

In addition, digital reconstruction of the damaged upper molars of *Plesiopithecus* (Fig. 4a) reveals similarities with two upper molars from Chamtwara that were previously identified as “Lorisidae indet.” by Harrison^23^ (Fig. 4b). Manipulation of digital surfaces of these upper molars allowed us to confirm that they occlude perfectly with *Propotto* lower molars from Chamtwara (Fig. 1e, f), and they are accordingly identified here as the first known upper teeth of *Propotto*. The morphology of these upper molars also resembles that of the possible stem lorisiform *Karanisia* from the earliest late Eocene of Egypt (Fig. 4d)^10^, the stem strepsirrhine *Djebelemur* from the early or middle Eocene of Tunisia^28^, and, intriguingly, the enigmatic late Eocene primate *Nosmips* from Egypt, which has been placed with *Plesiopithecus* in some phylogenetic analyses^20^. Among extant primates, *Propotto*’s upper molars (particularly M^1^) are most similar to those of the dwarf lemur *Cheirogaleus*. Similarities to *Djebelemur*, *Karanisia*, *Nosmips*, and *Plesiopithecus* include the broad lingual and more restricted buccal cingula, absence of a metaconule, and a concave distal margin of M^2^. Although some of these features may be primitive within Strepsirrhini, this character suite is nevertheless clearly characteristic of early strepsirrhines, and is not found in any living or extinct chiropteran. The upper molars of *Propotto* differ from those of known Paleogene strepsirrhines in being very low-crowned (matching the pattern seen in the lower molars), and in exhibiting a massive lingual cingulum, flattened lingual surfaces of the buccal cusps, and a reduced protocone.

### The anterior dentition of *Plesiopithecus*

**Fig. 5:**
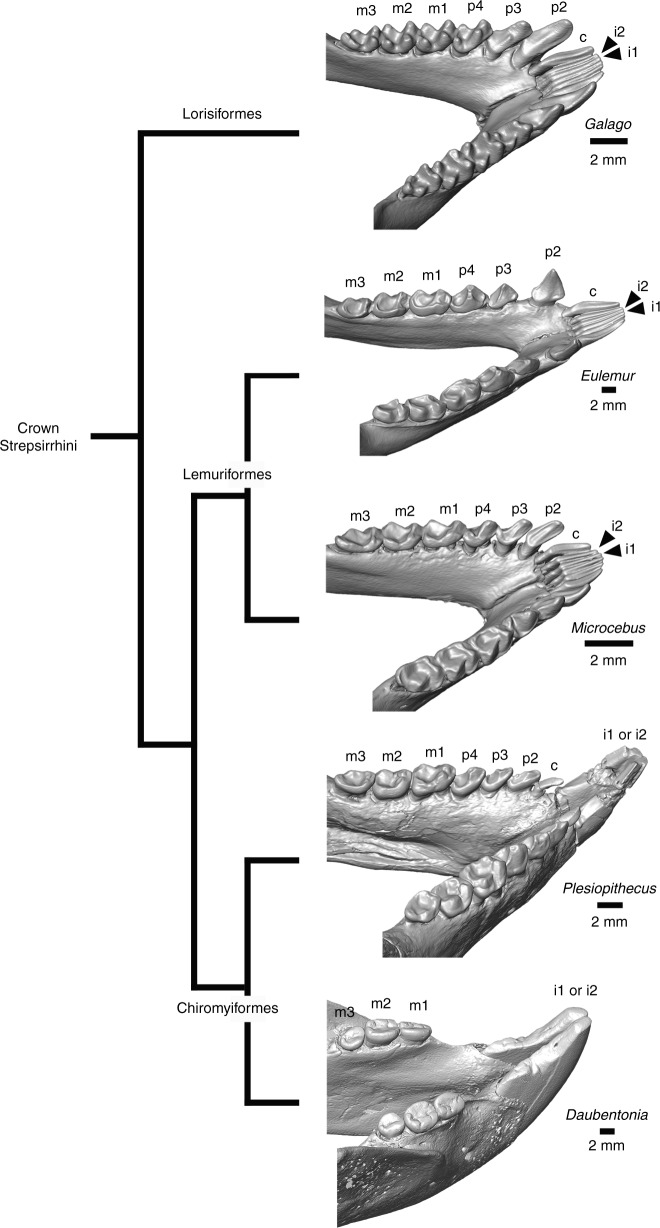
Comparative morphology of the lower dentition in crown strepsirrhines in phylogenetic context. From top to bottom, *Galago senegalensis* (MCZ 34381), *Eulemur fulvus rufus* (MCZ 16356), *Microcebus* (MCZ 45125), composite mandible of *Plesiopithecus teras* (DPC 11636; right side mirror-imaged), and *Daubentonia madagascariensis* (composite mandible using the corpus and incisor of AMNH M-185643 and the M_1–3_ of AMNH M-41334). Digital models were created using CT scans made available by the Museum of Comparative Zoology and Harvard University, the American Museum of Natural History, and the Duke Lemur Center Division of Fossil Primates, which were downloaded from www.morphosource.org and made available for reuse under a CC BY-NC license

The holotype of *Plesiopithecus teras* (CGM 42291; CGM = Egyptian Geological Museum) preserves a single enlarged and procumbent tooth mesial to P_2_-M_3_. A different mandibular specimen, DPC 11636 (DPC = Duke Lemur Center Division of Fossil Primates), was figured and discussed by Simons and Rasmussen^13^ (their Fig. 3) and preserves a small tooth (which the authors interpreted as a P_1_) between the enlarged anterior tooth and the P_2_. They did note, however, that the tooth “might also be the lateral canine derived from a toothcomb” (p. 9949). At some point after the description of this specimen in 1994, the crown of the tooth was broken and glued back onto the root, although rotated into an incorrect orientation. We have reconstructed the tooth using digital models and provide additional views of the specimen for the first time (Figs. 2c and 5). The tooth differs markedly in morphology and orientation from the adjacent P_2_, and has several features that are more consistent with it being a vestigial canine. The evolution of *Daubentonia*’s rodent-like incisor morphology from a toothcombed ancestor would likely involve topographically shifting the canine out of the toothcomb to accommodate an enlarged incisor. Indeed, in *Plesiopithecus*, this canine is strongly procumbent relative to its root, has a flattened surface (corresponding to the mesial face of a typical toothcomb canine, but more appropriately described as topographically lingual in DPC 11636) demarcated by a distinct ridge from the surface best exposed in occlusal view (lingual in a typical toothcomb canine, better described as topographically distal in *Plesiopithecus*), which has a gently curving and apically convex buccal margin. Morphological evidence supporting identification of this tooth as a lower canine rather than as a first premolar is supplemented by the dental formulae of all known living and extinct crown strepsirrhines, which unequivocally indicates that the loss of the upper and lower first premolar occurred along that clade’s stem lineage, and therefore before the appearance of both the strepsirrhine crown group and the split between the chiromyiform and lemuriform lineages. Indeed, no African strepsirrhine, living or extinct, is known to retain a P_1_. In light of this, it is more parsimonious to interpret this tooth of *Plesiopithecus* as a reduced lower canine, requiring the enlarged anterior tooth of *Plesiopithecus* to be an incisor, and therefore more likely homologous with the anterior tooth of *Daubentonia*. This enlarged anterior tooth also shows some thinning of the lingual enamel (relative to that on the buccal surface), though not to the extent seen in *Daubentonia*. The *Plesiopithecus* mandible DPC 13607 has also been digitally reconstructed, revealing a tiny canine alvelous anterior to the P_2_ (Fig. 1j); therefore the holotype is unlike the two other known specimens in lacking a canine.

### Phylogenetic placement of *Propotto* and *Plesiopithecus*

**Fig. 6:**
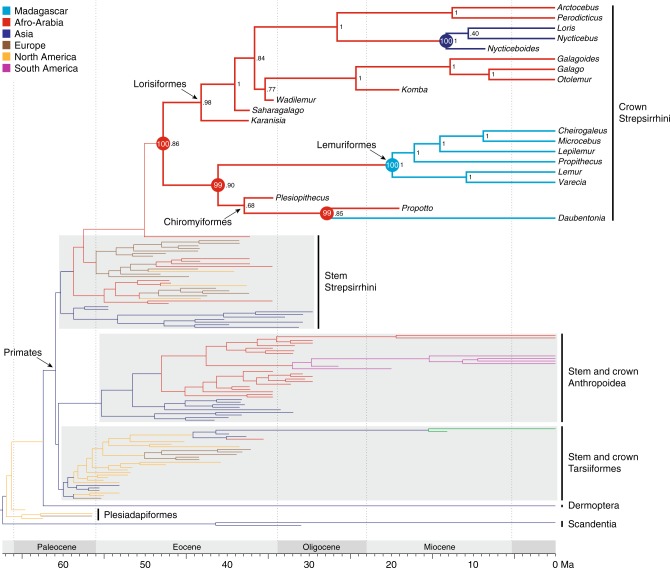
Phylogenetic relationships and biogeography of living and extinct strepsirrhines. Time-scaled tree derived from Bayesian tip-dating analysis of the combined molecular and morphological dataset. Terminal branches are color coded according to continental biogeography, and internal branches are color coded according to Bayesian ancestral biogeographic analysis. Numerical values to the right of nodes represent clade support (posterior probabilities) and circled numbers at each strepsirrhine node represent the posterior probability of each biogeographic reconstruction. Complete time-scaled phylogenetic trees and biogeographic reconstructions are available at the Dryad Digital Repository associated with this study (10.5061/dryad.gb182)

Phylogenetic analysis (see Methods) of our combined molecular and morphological data matrix using the Bayesian tip-dating method with fossilized-birth-death parameterization recovered *Propotto* as exclusively related to *Daubentonia*, and *Plesiopithecus* as the sister taxon to this *Daubentonia-Propotto* clade (Fig. 6). Standard Bayesian (“non-clock”) analysis recovered an exclusive *Propotto-Plesiopithecus* clade that is sister to *Daubentonia*. In both analyses, *Propotto* and *Plesiopithecus* are strongly supported as crown lemurs (posterior probability = 0.9) and are situated as stem chiromyiforms. Importantly, this result emerged despite controlling for two scoring biases that could have provided additional support for a *Daubentonia*-*Plesiopithecus*-*Propotto* clade. First, though there are sound reasons to consider *Propotto*’s enlarged anterior lower tooth to be homologous with that of *Plesiopithecus*, *Propotto* was conservatively not scored for either canine or incisor characters (i.e., only premolar and molar characters were scored). Second, *Plesiopithecus*’ enlarged anterior upper teeth were scored as canines and not incisors, although they could conceivably be enlarged incisors homologous with those of *Daubentonia*. To further avoid bias, we did not create any new characters or character states to capture novel observations of derived dental features shared by *Daubentonia* and *Propotto* to the exclusion of *Plesiopithecus* (see discussion below).

Bayesian stepping-stone estimation of marginal likelihoods for alternative placements of *Plesiopithecus* and *Propotto*, using the morphology matrix and constraining these two taxa to fall in different positions within the optimal time-scaled tree derived from the tip-dating analysis of molecular and morphological data, reveals that there is “strong” evidence (based on a Bayes factor of 15.64) for favoring a (*Plesiopithecus* (*Daubentonia*, *Propotto*)) topology over the (*Daubentonia* (*Plesiopithecus*, *Propotto*)) topology derived from the non-clock analysis. Other alternative constraints, such as situating *Plesiopithecus* and *Propotto* as advanced stem strepsirrhines or as stem lemurs, were decisively rejected by stepping-stone analyses (based on Bayes factors of 572.49 and 651.97, respectively). Bayesian reconstruction of ancestral morphological character states on the optimal clock topology identified 15 character state changes along the chiromyiform stem leading to the (*Plesiopithecus* (*Daubentonia*, *Propotto*)) clade, and 18 character state changes along the lineage leading to the *Daubentonia*-*Propotto* clade. The monophyly of Eocene-Recent chiromyiforms is supported by character changes relating to the modification of the anterior dentition to include only a single enlarged and procumbent incisor, as well as numerous details of premolar and molar crest and cusp development/placement, and increased depth of the mandibular corpus (see supporting data files held in the Dryad Digital Repository associated with this study (10.5061/dryad.gb182)).

### Biogeographic history of lemurs

Bayesian reconstruction of strepsirrhine biogeographic history strongly supports (posterior probability = 1) African origins for both Chiromyiformes and Lemuriformes, implying independent dispersals across the Mozambique Channel. Our analyses place the last common ancestor of *Daubentonia* and *Propotto* on the African continent at 27.9 Ma (near the early-late Oligocene boundary), suggesting that the dispersal to Madagascar that ultimately gave rise to *Daubentonia* likely occurred some time after the early Oligocene. The continental divergence of the chiromyiform and lemuriform lineages is estimated at 41.1 Ma (late middle Eocene) and the island origin of crown lemuriforms is estimated at 19.9 Ma (early Miocene).

## Discussion

Our analyses suggest that *Propotto* and *Plesiopithecus* are stem chiromyiform lemurs that are closely related to the extant aye-aye *Daubentonia* from Madagascar, and that stem chiromyiforms were present in Africa from at least the late Eocene through the early Miocene. Our time-scaled tree, when combined with Bayesian biogeographic analyses, strongly supports an African origin for the common ancestor of lemuriforms and chiromyiforms, and independent dispersals of these groups across the Mozambique Channel to Madagascar. Although independent African origins for two closely related Madagascan lineages might appear overly coincidental, recent molecular phylogenies of chameleons indicate that there were two independent colonizations of Madagascar by African lineages in the Cenozoic^29^, establishing an independent precedent for the feasibility of such a pattern. Our results suggest that the chiromyiform dispersal to Madagascar occurred no earlier than the Oligocene (based on the divergence date of *Propotto* and *Daubentonia*), while the lemuriform dispersal could have occurred no later than the early Miocene (based on the time of origin of the crown lemuriform clade). Our results do not allow us to address the question of when the lemuriform dispersal to Madagascar occurred within the ~41 to ~20 Ma dispersal window provided by our analyses, but it is noteworthy that the combined phylogenetic and biogeographic evidence can now accommodate a scenario in which lemuriforms dispersed to Madagascar quite late in the Cenozoic (i.e., as late as the earliest Miocene), where they then underwent an adaptive radiation. Because our results do not require lemuriforms to have been present on Madagascar until the early Miocene, they render the recently proposed hypothesis of a mass extinction of lemuriforms on Madagascar near the Eocene–Oligocene boundary^1^ less likely, but not impossible.

Previously, lemurs have been regarded as the first placental mammals to colonize Madagascar (with the possible exception of the enigmatic subfossil mammal *Plesiorycteropus*, whose affinities are uncertain). However, our results suggest much later dispersal windows for lemuriforms and chiromyiforms which overlap with those that have been recently estimated for Madagascar’s other endemic terrestrial mammals—i.e., euplerid carnivorans^30,31^, nesomyine rodents^31^, and tenrecids^31,32^. The Oligocene to early Miocene interval also overlaps with recently proposed dispersal windows for hyperoliid frogs^8,33^, lamprophiid snakes^8,34^, zonosaurine lizards^8,35^, as well as multiple scincids and gekkonids^8^. Importantly, this interval was also characterized by the lowest sea levels in the Cenozoic^36^, prior to the onset of middle Miocene cooling.

Several additional derived dental features shared by *Propotto* and *Daubentonia* (to the exclusion of *Plesiopithecus*) would be consistent with a close relationship of the former two genera, but were not captured by the morphological matrix used here. In the upper dentition, both taxa have particularly well-developed parastyle and metastyle cusps that are close in height to the paracone and metacone, respectively; flat (as opposed to convex) lingual surfaces of the buccal cusps; and low molar protocones. In the lower dentition, *Daubentonia* and *Propotto* share very shallow talonid basins; tall and wall-like oblique cristids; and highly reduced (*Propotto*) or absent (*Daubentonia*) metaconid and entoconid cusps, protocristid crests, and hypoflexids on M_1–2_. The *Propotto* specimen KNM-RU 3690 shows that the mesial aspect of P_3_ was much closer to the alveolus for the enlarged anterior lower tooth than in *Plesiopithecus*, suggesting that the P_2_ of *Propotto* was smaller than that of *Plesiopithecus* and that there was no room for a canine as in *Plesiopithecus*; this pattern is derived toward the total loss of the lower premolars seen in *Daubentonia*.

From a functional perspective, the distally oriented postprotocrista and mesiodistally aligned lingual cingulum on the M^1^ of *Propotto*, combined with the low M^1–2^ protocones, flattening of the talonid basins and reduction of the hypoflexids, protocristids, and metaconids on the lower molars (features not present in *Plesiopithecus*) reflects increased emphasis on propalinal mastication and associated development of predominantly mesiodistally aligned upper and lower molar wear facets, as in *Daubentonia*. If *Propotto* is indeed a close relative of *Daubentonia*, the former’s massive lingual cingula and reduced protocone and metaconid cusps might even help to explain the strange lingual region of M^1–2^ in *Daubentonia*. These teeth superficially appear to lack a lingual cingulum, but nevertheless bear hypocone cusps, structures that are always derived from the distolingual cingulum in crown strepsirrhines. The fact that the M^1–2^ hypocones in *Daubentonia* are connected buccally to the postmetacristae by what appear to be tall and thick postcingula, and are continuous mesiolingually with similarly tall and thick elongate ridges, hints at the possibility that the latter features might be derived from the lingual cingulum and not the protocone, and that the protocones (which are highly reduced in *Propotto*) are effectively absent in *Daubentonia*. A possible mechanism for this transformation is provided by the occlusal morphology of the *Propotto* molars from Chamtwara, the M_1_ of which bears a cingulid lingual to the very reduced metaconid that occludes with the lingual cingulum of M^1^. Digital manipulation of these surfaces indicates that the gutter between the mesial aspect of the M^1^ lingual cingulum and the protocone occludes on top of the reduced M_1_ metaconid, which is a remarkably odd arrangement, but one that, taken to an extreme, would yield a morphology like that seen in *Daubentonia* (Fig. 2a). The morphological similarity of *Daubentonia* and *Propotto* M_2_ surfaces, as supported by our automated geometry analyses, lends further credibility to this hypothesis. Again, these novel interpretations of the cusp/crest homologies of *Daubentonia* were not scored as characters in our phylogenetic analyses to avoid circularity in our assessment of phylogenetic relationships of the taxa in question.

Marked restructuring of interpretations of strepsirrhine biogeographic history suggested by our analyses presently depends almost exclusively on dental morphology, hence more rigorous tests of these hypotheses will only be possible as new and more complete fossils are discovered. An obvious challenge to the hypothesis that chiromyiforms and lemuriforms independently dispersed to Madagascar is the current lack of diagnostic stem lemuriforms in the African fossil record. Notably, the Paleogene fossil record of Afro-Arabia is notoriously poor and geographically biased toward northern Africa^37^. Nonetheless, a handful of fragmentary fossils provide tantalizing evidence of possible lemuriform-like strepsirrhines from other parts of Afro-Arabia, such as *Notnamaia* from Namibia^38^, and *Omanodon* and *Shizarodon* from Oman^39^. Furthermore, an early Miocene origin for the crown lemuriform clade does not necessarily require that the Africa-to-Madagascar dispersal event be coincident with, or even close in age to, the origin of that clade—that dispersal could have occurred at any point along the long lemuriform stem lineage. Therefore, absence of lemuriform fossils from the Paleogene of Afro-Arabia might be explained by an early dispersal to Madagascar closer to the chiromyiform–lemuriform split, followed by the extinction of basal stem lemuriforms in Africa. An analogous pattern is seen in the fossil record of platyrrhine anthropoid primates that likely also originated in Afro-Arabia and then dispersed to South America, from an as-yet unsampled stem lineage in Afro-Arabia.

Regardless of the phylogenetic and biogeographic history of *Daubentonia*, it is of great paleoecological significance that Cenozoic African primate communities gave rise to a somewhat *Daubentonia*-like (and presumably tree gouging) primate lineage, as occurred in various non-primate mammalian radiations on other continents, such as Apatemyidae and various plesiadapiform euarchontans on northern continents, and the marsupial groups Petauridae and Yalkaparidontia in Australasia^40,41^. It is also significant that the strepsirrhine lineage represented by *Plesiopithecus* and *Propotto* persisted in Africa well into the Miocene, long after major perturbations in Earth climate history such as global cooling at the Eocene–Oligocene boundary and biotic events in Africa such as the immigration of multiple mammalian lineages in the late Oligocene or early Miocene. These patterns add to the evidence that equatorial Africa likely had a key role as a relatively temperate refugium for primate communities at a time of marked mid-Cenozoic ecological restructuring^42,43^.

## Methods

### Phylogenetic analysis

We tested Simpson’s original hypothesis of *Propotto*-lorisid affinities, and Godinot’s hypothesis of a *Daubentonia-Plesiopithecus* clade within Strepsirrhini, by adding *Daubentonia* and *Propotto* to an augmented version of Seiffert et al.’s^44^ morphological character matrix, rescoring the anterior lower dentition of *Plesiopithecus* (taking into account the observations detailed above), and combining those morphological data with the molecular dataset of Springer et al.^4^. The dermopteran *Galeopterus* was also added to the matrix and scored for morphological characters, so that two extant euarchontan outgroup taxa were scored for both molecular and morphological data; the augmented morphological dataset now includes 102 fossil and 23 living euarchontans and 395 characters. To maximize phylogenetic signal, 264 ordered characters were constructed using intermediate states to code for polymorphic observations^45^. However, MrBayes currently imposes a six-state limit for ordered transformation series, necessitating that 39 of these characters be recoded using standard polymorphic scoring.

The DNA supermatrix of Springer et al.^4^ consists of 69 nuclear and 10 mitochondrial gene segments totaling 61,119 positions. To optimize data completeness, molecular sequences for *Lepilemur ruficaudatus* and *Propithecus verreauxi* were selected to accompany morphological character scores for their respective genera. All other taxa were scored for both molecular and morphological data at the species level, and species present in the DNA supermatrix but absent from the morphological dataset were removed. PartitionFinder v2.1.1^46^ was used to select a DNA subset scheme and nucleotide substitution models. Each gene segment was assigned as an input block and testing included all models available in MrBayes. The best parameterization was assessed using the Bayesian Information Criterion (BIC), which recommended a scheme with 13 subsets and a combination of General Time Reversible (GTR + G and GTR + I + G) models.

The molecular and morphological datasets were concatenated with Mesquite v3.20^47^. We used the parallel (MPI) version of MrBayes v3.2.6^48^ to conduct two total-evidence Bayesian phylogenetic analyses. The first was a standard “non-clock” analysis. The second was a time-scaled “clock” analysis which implemented the tip-dating method with fossilized-birth-death (FBD) parameterization. For the molecular portion of the matrix, settings for partitions and models were assigned based on the PartitionFinder results. The morphological portion of the matrix was set as a single partition using a gamma-distributed Markov *k* model and variable coding to accommodate ascertainment bias. Parameters for models across all 14 partitions were set as unlinked. Two hard topological constraints were applied—one enforced monophyly for primates and the other constrained scandentians as an outgroup. Metropolis coupled Markov chain Monte Carlo (MCMCMC) parameters were set for 2 runs with 4 chains each and to sample in 1000 generation increments. To promote chain swapping, the heating temperature was set to 0.02.

Run length was assessed and chosen using built-in MrBayes diagnostics for convergence and sampling sufficiency. First, we targeted run lengths yielding an average standard deviation of split frequencies (ASDSF)≤0.01. The value of this diagnostic should approach zero as individual runs converge on topological distributions. Second, we targeted run lengths in which the minimum estimated sampling size (minESS) of all parameters was ≥ 100. Burn-in settings were selected to optimize these two diagnostics. We explored absolute burn-in generation values in increments of 5 M, ranging from 5 M up to 50% of run lengths. At each increment, tree and parameter summarizations were run and diagnostic data were collected. The final burn-in value for each analysis was selected by identifying all values that yielded the ASDSF target (≤0.01), and then from this group, choosing the one which yielded the minESS set whose least sampled parameter most exceeded the minESS target (≥100). Under this run-length strategy, the non-clock analysis was run for 92 M generations of which 5 M were discarded as burn-in. The resulting ASDSF was 0.00995 and the smallest minESS in the parameter set was 532.5906. The tree distribution was summarized using the “allcompat” (majority-rule plus compatible groups) option.

Our clock analysis included several additional settings. (1) The node age prior was set as calibrated with living taxa fixed to zero (=present day) and fossil taxa constrained to an age range. Age ranges were estimated using (when possible) the currently recognized upper and lower bounds of magnetochrons, “land mammal ages”, and/or other radiometric constraints in which each fossil taxon may be reasonably placed^44^. Fossil-tip ages were set to sample from uniform distributions across these ranges. (2) For the clock variance prior, we used the independent gamma rates (IGR) model to estimate relaxed clock rates. For the associated IGR variance prior, we used the default MrBayes setting (exponential distribution, *λ* = 10). (3) We applied the FBD model for the prior probability distribution of branch lengths and used the fossil-tip sampling strategy. The FBD extinction and fossilization priors were set as flat (beta distributions, *α* = 1, *β* = 1) and the FBD speciation prior was set to the MrBayes default (exponential distribution, *λ* = 10). The FBD model also requires a sampling probability prior which is an estimate for the proportion of extant taxa included in the study. With about 450 living primate species^4^, 2 currently recognized living dermopteran species^49^ (though it should be noted that there is now strong evidence for cryptic diversity within Demoptera^50^), and 20 living scandentian species^51^, the 23 extant taxa in our matrix constitute ~0.0487 of living euarchontan diversity. (4) The clock rate prior is an initial estimate for a distribution describing the number of substitutions per site per million years. To derive this prior, we used novel R code that utilizes the non-clock tree, age estimates for each taxon and an age estimate for the tree root. First, the dist.nodes function from the R package APE v3.4^52^ was used to extract path lengths from each tree tip to the tree root. Next, each path length was scaled by the difference between the root age estimate and a tip age estimate. For the root age, we used 65.2 Ma that corresponds to the earliest bound for *Purgatorius*, the oldest fossil taxon in the dataset. For tip ages, we used zero for living taxa and age range midpoints for fossil taxa. Finally, the fitdist function from the R package fitdistrplus v1.0-6^53^ was used to fit normal, lognormal and gamma distributions to the set of scaled path lengths. The best model was assessed using the BIC, in this case a lognormal distrbution with a mean of −3.983465721 and a standard deviation of 0.564231504. This model and its parameter values were used directly for the MrBayes clock rate prior. (5) Age calibrations were applied to both the Primates and Euarchonta nodes. Calibration settings specified truncated normal distributions with minimum and mean ages corresponding to the earliest bound of the oldest fossil taxon in the group (Primates: *Teilhardina* = 55.8 Ma, Euarchonta: *Purgatorius* = 65.2 Ma). Pre-Eocene ghost lineages were penalized by setting 1 Ma standard deviations on these distributions. The clock analysis was run for 150 M generations, of which 20 M were discarded as burn-in. The resulting ASDSF was 0.009053 and the smallest minESS in the parameter set was 138.0835. The tree distribution was summarized using the “allcompat” option.

### Comparing likelihoods of alternative topologies

Both the clock and non-clock analyses recovered stem chiromyiform positions for *Plesiopithecus* and *Propotto*, but with different placements relative to *Daubentonia*. As time is a fundamental factor in estimating branch lengths of phylogenetic trees, and statistical methods such as Bayesian inference inseparably consider branch lengths and topology in likelihood calculations, we regard the result of the clock analysis as optimal. To compare the estimated marginal likelihood of the clock result to those of alternate topologies, we conducted a set of post hoc analyses using MrBayes. To do so, *Plesiopithecus* and *Propotto* were pruned from the time-scaled clock tree, and the remaining tree was used as a soft constraint for reanalysis of the morphological partition with stepping-stone sampling. (A) The first reanalysis allowed *Plesiopithecus* and *Propotto* to be placed anywhere in the tree. The result was identical to the clock analysis—specifically that *Plesiopithecus* is positioned as sister to a *Propotto*-*Daubentonia* clade [H1, optimal]. (B) The second reanalysis disallowed a *Propotto*-*Daubentonia* group. The resultant topology for Chiromyiformes was identical to the non-clock analysis—specifically that a *Propotto*-*Plesiopithecus* clade is positioned as sister to *Daubentonia* [H2]. Comparison of the estimated marginal likelihood of H1 to that of H2 yielded a Bayes factor of 15.6. (C) The third reanalysis disallowed placements of *Plesiopithecus* and *Propotto* as stem chiromyiforms. Given this constraint, a *Propotto*-*Plesiopithecus* clade was instead positioned as the immediate sister clade of crown strepsirrhines [H3]. Comparison of the estimated marginal likelihood of H1 to that of H3 yielded a Bayes factor of 572.5. (D) The fourth reanalysis also disallowed placements of *Plesiopithecus* and *Propotto* as stem chiromyiforms, but forced these taxa to be placed within crown Strepsirrhini. Given these constraints, a *Propotto*-*Plesiopithecus* clade is positioned as the immediate sister group of the chiromyiform–lemuriform clade [H4]. Comparison of the estimated marginal likelihood of H1 to that of H4 yielded a Bayes factor of 652.0. We interpret Bayes factors >10 as strong evidence, and >100 as decisive evidence, in favor of the optimal hypothesis versus alternate hypotheses^54,55^.

### Morphological character state transformations

MrBayes was used to conduct ancestral state reconstructions (ASR) for all morphological characters that were scored for either *Plesiopithecus* or *Propotto* (*n* = 166). Given the topology and branch lengths of the “allcompat” tree derived from the clock analysis, ASR provides the probabilities of all states of all characters for each ancestral node. For each character, we considered the state with the highest probability to be the best estimate at an ancestral node, provided that the probability of that state exceeded all others by >10%. If the probability of a runner-up state was ≤10% of the highest, we considered the best ancestral estimate to be inclusively polymorphic.

### Biogeographic analysis

Geographic distribution for each taxon in the dataset was coded as one of the following six land masses: North America, South America, Europe, Asia, Afro-Arabia, Madagascar. For both the optimal [H1] and first runner-up [H2] trees, MrBayes was used to conduct ASR of biogeography; both analyses support independent dispersals of the chiromyiform and lemuriform lineages from Afro-Arabia to Madagascar. A concise summary of ancestral biogeography on the optimal tree is presented in Fig. 6.

### Auto3dgm analysis of tooth shape

To assess the phenetic affinities of the molar teeth of *Propotto* in an objective and quantitative way, we used an automated three dimensional (3D) geometric morphometric analysis. Our sample consists of lower second molars of 222 individuals representing 42 genera (Supplementary Data 1). Digital models of these specimens were created by micro-CT scanning physical specimens (either casts or originals); Avizo versions 6-8.1^56^ were used to fit and crop surfaces, followed in some cases by further processing (patching and smoothing) in Geomagic^57^. Euarchonta is comprehensively represented (with the exception of anthropoids), including a diversity of early fossil prosimians. We also include molars of two pteropodid genera, as a test of the hypothesis that *Propotto* is a primitive fruit bat. Automated analyses allow an objective and comprehensive representation of shape. The software evenly spreads a user-specified number of “pseudolandmarks” over the surface of a 3D object and then algorithmically determines the correspondence among landmarks on different bones^58^. We used the MATLAB version of auto3dgm, the most recent version of which can be accessed by contacting the authors or through the github address: https://github.com/trgao10/PuenteAlignment/. We used the following parameters in our analysis: 300 initial pseudolandmarks, 1100 final pseudolandmarks, 3000 iterations of iterative closest points, and reflections allowed. The analysis was run on the mathematics computing cluster at Duke University. The Procrustes transformed pseudolandmark coordinates are available (Supplementary Data 1). The aligned coordinates of the pseudolandmarks from Table S1 were then analyzed using principal components analysis of tangent space in *morphologika*^2.5^ (Supplementary Data 2 and 3). We decided to remove *Prolemur* from the analysis post hoc given the small sample (*n* = 2), and problems with breakage on one tooth and heavy wear on the other. Additionally, we identified seven outlier specimens (in the sense that they plotted far from other individuals of their species) in an early version of the analysis. These seven outliers turned out to have mesh or alignment issues as noted in the footnote of Supplementary Data 1.

### Data availability

Input data files, settings, and results from phylogenetic, stepping stone, ASR, and biogeographic analyses are available on the Dryad Digital Repository (10.5061/dryad.gb182). Digital surface models for all of the figured fossil specimens are available on MorphoSource (www.morphosource.org, see Table 1 for digital object identifiers).

## Electronic supplementary material

Supplementary Information

Description of Additional Supplementary Files

Supplementary Data 1

Supplementary Data 2

Supplementary Data 3
