# Supplementary material for: Fossil lemurs from Egypt and Kenya suggest an African origin for Madagascar’s aye-aye
Source: Nat Commun. 2018 Aug 21;9:3193. doi: 10.1038/s41467-018-05648-w (PMC6104046; doi:10.1038/s41467-018-05648-w)
Supplement: Supplementary file 2 — Description of Additional Supplementary Files [file 41467_2018_5648_MOESM2_ESM.pdf]

Description of Additional Supplementary Files

File Name: Supplementary Data 1
Description: Coordinates of 1,100 Procrustes aligned pseudolandmarks for 222 euarchontans (without foot note on outliers).

File Name: Supplementary Data 2
Description: Principal component scores of 213 euarchontans derived from 1,100 pseudolandmarks of Supplementary Data 1.

File Name: Supplementary Data 3
Description: Eigenvalues and % variance for PCA on 1,100 pseudolandmarks.
