# Supplementary material for: Fossil lemurs from Egypt and Kenya suggest an African origin for Madagascar’s aye-aye
Source: Nat Commun. 2018 Aug 21;9:3193. doi: 10.1038/s41467-018-05648-w (PMC6104046; doi:10.1038/s41467-018-05648-w)
Supplement: Supplementary file 1 — Supplementary Information [file 41467_2018_5648_MOESM1_ESM.pdf]

Supplementary Information for Gunnell *et al*. "Fossil lemurs from Egypt and Kenya suggest an African origin for Madagascar's aye-aye"

Contents: Supplementary Figure 1

MUSEUM OF COMPARATIVE ZOOLOGY

The Agassiz Museum

HARVARD UNIVERSITY · ~~CAMBRIDGE, MASSACHUSETTS~~ 02138 · TEL. 617 868-7600

Tucson, Ariz.

Dear Dr. Walker:

Lewis Leakey has forwarded to me the copy of your paper on <u>Propotto</u> that you sent to him.

I have studied this carefully, & since doing so I have slept little & worked not at all. This of course is my own fault, not yours. Certainly I have been mistaken before, but this is the blunder for which I can least excuse myself.

– But that need hardly concern you. I only mean to say that I agree with you, & also agree that correction must be published promptly.

Sincerely,

G. G. Simpson

Dear Dr. Walker:

Lewis(sic) Leakey has forwarded to me the copy of your paper on *Propotto* that you sent to him.

I have studied this carefully, & since doing so I have slept little & worked not at all. This of course is my own fault, not yours. Certainly I have been mistaken before, but this is the blunder for which I can least excuse myself.

- But that need hardly concern you. I only mean to say that I agree with you, & also agree that correction must be published promptly.

Sincerely,

G.G. Simpson

Supplementary Figure 1. Letter from George Gaylord Simpson to Alan Walker regarding the affinities of *Propotto* (no date).
